# [Corrigendum] Hsp90 inhibitor 17‑AAG inhibits stem cell‑like properties and chemoresistance in osteosarcoma cells via the Hedgehog signaling pathway

**DOI:** 10.3892/or.2023.8593

**Published:** 2023-06-21

**Authors:** Xiong Shu, Huiqi Liu, Rui Zhen, Yongsheng Jie, Lei Chen, Hui Qi, Chao Wang, Renxian Wang, Dafu Chen, Yuliang Ran

Oncol Rep 44: 313–324, 2020; DOI: 10.3892/or.2020.7597

Following the publication of the article, a concerned reader drew to the authors' attention that, in Fig. 1B and C on p. 316, two pairs of the data panels showing the results from invasion and migration assay experiments appeared to be overlapping, such that they would have been derived from the same original sources where they were intended to show the results from different experiments; moreover, on p. 1698, the ‘17-AAG / MG-63’ data panels in Fig. 3B and C were also overlapping, albeit the images were presented at a different scale and in a slightly different orientation.

After having examined their original data, the authors have realized that these figures were inadvertently assembled incorrectly. The corrected versions of Figs. 1 and 3, now showing the correct data in Fig. 1C (where the errors made in compiling the figure had occurred) and the correct data for the ‘17-AAG / MG-63’ data panel in Fig. 3C, are shown on the next two pages. These corrections do not grossly affect either the results or the conclusions reported in this work. The authors all agree to the publication of this Corrigendum, and are grateful to the Editor of *Oncology Reports* for granting them the opportunity to correct the errors that were made during the assembly of these figures. Lastly, the authors apologize to the readership for any inconvenience these errors may have caused.

## Figures and Tables

**Figure 1.**
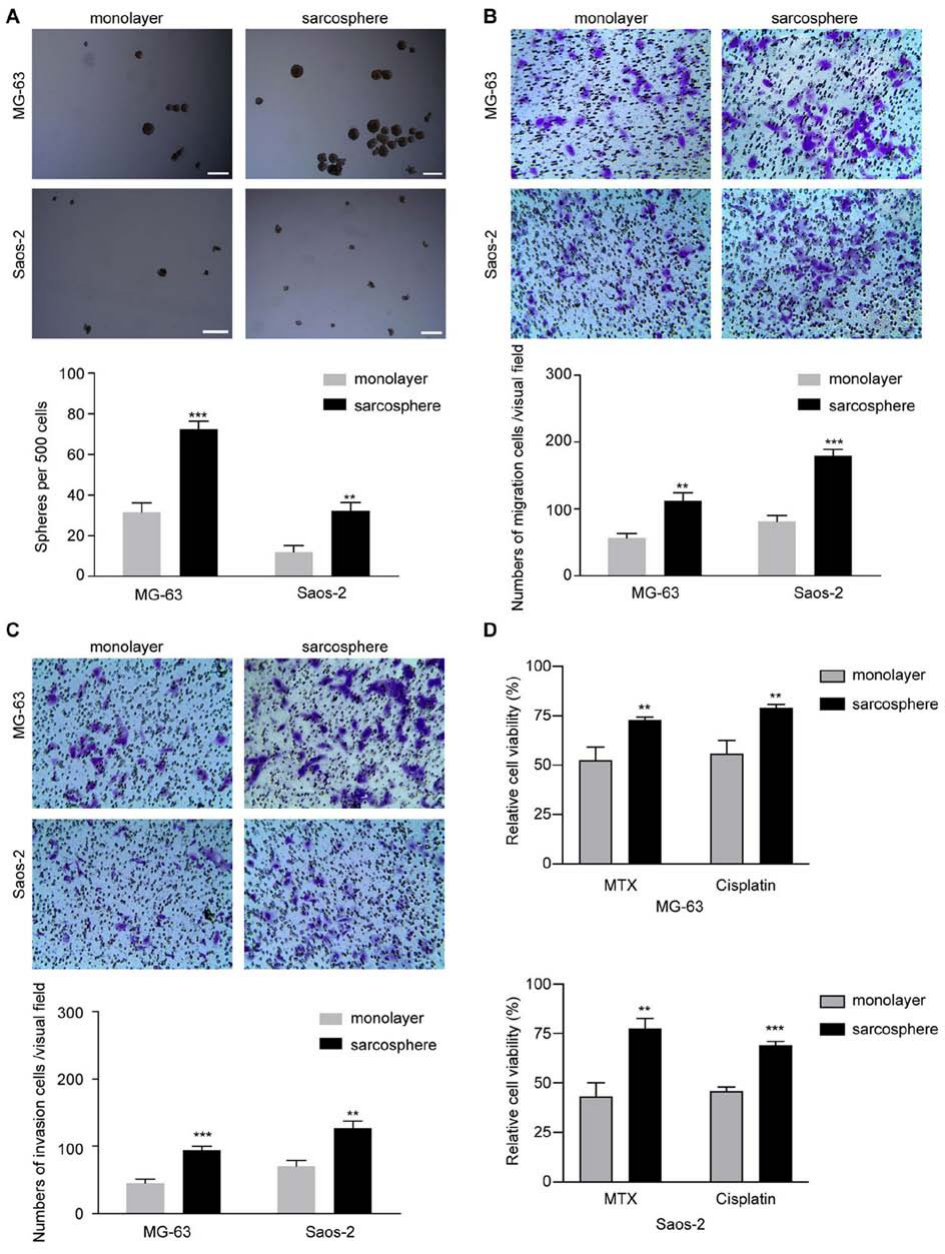
Osteosarcoma sarcosphere cells exhibit stem-like properties and chemotherapy resistance. (A) The spheroid-forming abilities of sarcosphere and monolayer cells derived from the MG-63 and Saos-2 cell lines were determined using low-attachment plates during methylcellulose culture Scale bar, 50 µm. (B) The migratory capacity of sarcosphere and monolayer cells derived from the MG-63 and Saos-2 cell lines was determined using a Transwell migration assay. (C) The invasive capacity of sarcosphere and monolayer cells derived from the MG-63 and Saos-2 cell lines was determined using a Matrigel invasion assay. (D) Treatment with methotrexate or cisplatin chemotherapy compared with DMSO. Data are presented as the mean ± standard deviation of three independent experiments. **P<0.01, ***P<0.001. MTX, methotrexate.

**Figure 3.**
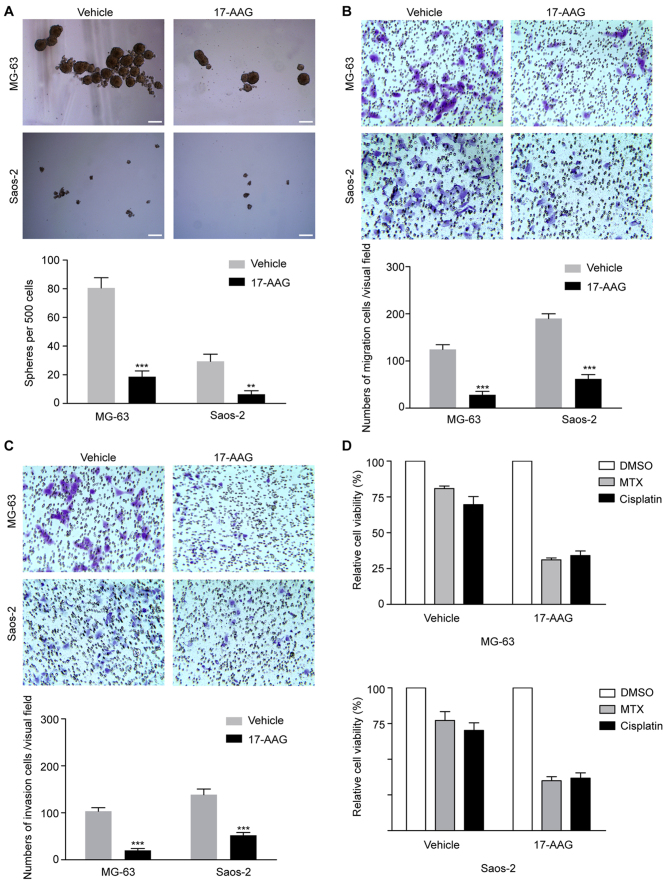
17-AAG suppresses stem cell-like properties and chemoresistance of osteosarcoma sarcosphere cells. (A) The spheroid-forming abilities of sarcosphere cells derived from the MG-63 and Saos-2 cell lines were analyzed following treatment with 50 nM 17-AAG using low-attachment plates during methylcellulose culture. Scale bar, 50 µm. (B) The migration capacity of sarcosphere cells derived from MG-63 and Saos-2 cells was determined in 17-AAG-treated cells using a Transwell migration assay. (C) The invasion capacity of sarcosphere cells derived from the MG-63 and Saos-2 cell lines following treatment with 17-AAG was determined using a Matrigel invasion assay. (D) Treatment with methotrexate or cisplatin chemotherapy compared with DMSO following treatment with 17-AAG. Data are presented as the mean ± standard deviation of three independent experiments. **P<0.01, ***P<0.001.

